# Single-season responses of rhizosphere fungal community structure and diversity to combined application of different organic materials in potato

**DOI:** 10.3389/fmicb.2026.1853956

**Published:** 2026-09-24

**Authors:** Lianglu Hao, Xinyuan Hu, Bo Dong, Shiqian Guo, Dandan Du, Li Jia, Jian Wen

**Affiliations:** 1 College of Resources and Environment, Gansu Agricultural University, Lanzhou, China; 2 Dryland Agriculture Institute, Gansu Academy of Agricultural Sciences, Lanzhou, China; 3 Key Laboratory of Efficient Utilization of Water in Dry Farming of Gansu Province, Lanzhou, China; 4 Gansu Provincial General Station for Cultivated Land Quality Construction and Protection, Lanzhou, China

**Keywords:** fungal community structure, fungal diversity, organic materials, potato, rhizosphere soil

## Abstract

Long-term application of chemical fertilizers can lead to a decline in soil microbial diversity and an imbalance in community structure, thereby constraining the sustainable development of the potato industry. In this study, a single-season field experiment was conducted in Yongdeng County, Lanzhou City, Gansu Province, with four treatments: chemical fertilizer alone (CF), sheep manure combined with chemical fertilizer (T1), biochar combined with chemical fertilizer (T2), and corn stover combined with chemical fertilizer (T3). High-throughput sequencing was employed to investigate the single-season responses of fungal community structure and diversity in potato rhizosphere soil to different combined application patterns of organic materials and chemical fertilizers. The results showed that: (1) compared with CF, the T1, T2, and T3 treatments significantly increased soil organic matter, available phosphorus, and total nitrogen contents, but had no significant effects on soil pH, total phosphorus, or total potassium. Crop yield was significantly increased in the T1 and T2 treatments compared with CF, whereas no significant difference was observed in the T3 treatment. (2) Compared with CF, the Chao index was significantly increased in the T2 and T3 treatments, and the Shannon index was significantly increased in the T1, T2, and T3 treatments. Beta diversity analysis revealed that the T1, T2, and T3 treatments all induced significant changes in fungal community structure, with T1 and T3 exhibiting relatively high similarity in community composition. (3) Compared with CF, the T3 treatment significantly increased the relative abundances of *Alternaria* and *Mortierella* while decreasing that of *Pseudogymnoascus*; the T2 treatment significantly increased the relative abundance of *Mortierella* and decreased that of *Pseudogymnoascus*; and the T1 treatment significantly increased the relative abundance of *Scutellinia*. (4) LEfSe analysis revealed that the T2 treatment harbored the highest number of differentially abundant fungal taxa (8 species). Redundancy analysis identified soil total nitrogen as the most important environmental driver shaping fungal community variation. In summary, under the single-season conditions of this experiment, the T2 treatment exhibited the best performance in enhancing both potato yield and fungal diversity; however, its long-term effects require further validation.

## 1 Introduction

The potato (*Solanum tuberosum* L.) is the fourth most widely cultivated food crop worldwide and plays a vital role in ensuring global food security and promoting agricultural development and economic growth (Gómez et al., 2019). As one of China’s major potato-producing regions, Gansu Province offers natural advantages for the development of the potato industry due to its unique climatic conditions and soil environment (Pan et al., 2025). In 2022, the province’s potato cultivation area reached 574,700 hectares, with a total output of 15 million tons, ranking third nationally in both cultivation area and output (Qi, 2023). However, as potato cultivation areas continue to expand and intensive production models are promoted, the sustainable use of soil resources is under threat. The long-term application of chemical fertilizers has led to the deterioration of soil physicochemical properties and a decline in soil fertility (Liu, 2018). It has also caused an imbalance in the structure of soil microbial communities, reducing the stability and functionality of the soil ecosystem. This has exacerbated the accumulation of pathogens, increased the risk of soil-borne diseases, and seriously threatened potato yield and quality (Gao et al., 2023; Liu X. et al., 2016).

As a vital component of the soil microbial community, soil fungi play a significant role in maintaining soil quality and productivity by decomposing plant and animal residues, accumulating organic matter, and suppressing pests and diseases (Clemmensen et al., 2013). Soil fungi can also form symbiotic mycorrhizal associations with crops, promoting plant growth and maintaining the stability of agricultural ecosystems (Brito et al., 2012). Therefore, maintaining a stable structure and rich diversity of fungal communities is of great significance for guiding sustainable agricultural production in the region. Studies have shown that the application of organic materials can improve soil physical and chemical properties and modulate microbial community structure, thereby driving the succession of the soil microecosystem toward a healthy and stable state (Ma et al., 2021; Yang K. et al., 2024). Agricultural and livestock byproducts such as corn stover and sheep manure are rich in organic matter and are commonly used for soil improvement. Due to differences in their carbon-to-nitrogen ratios and physical properties, their effects on soil nutrients and fungal communities also vary. An et al. (2022) found that the application of sheep manure organic fertilizer in combination with chemical fertilizers can enhance the availability of soil nutrients in lychee orchards, increase microbial diversity, alter the composition of microbial communities, and ultimately establish a microbial community more conducive to crop growth. Li et al. (2026) found that straw return increased soil fungal diversity while reducing the abundance of *Plectosphaerella*. Ma et al. (2021) found that available phosphorus, pH, and the carbon-to-nitrogen ratio are key factors regulating the structure of nitrogen-fixing microbial communities. Biochar, characterized by its highly aromatic structure and strong chemical stability, is commonly used as a soil amendment. Chang et al. (2024) found that the application of biochar increased fungal richness and diversity, promoted the proliferation of *Mortierella* and *Rhizophlyctis*, and inhibited the proliferation of *Fusarium*.

Although previous studies have investigated the effects of organic material application on soil fungal communities, field research remains limited in the arid and semi-arid regions of Northwest China regarding how the combined application of organic materials with markedly different carbon-to-nitrogen ratios and physical properties affects the potato rhizosphere fungal community. To address this gap, a single-season field experiment was conducted in the potato-producing area of Yongdeng County, Gansu Province. Sheep manure, biochar, and maize straw were selected as representative organic materials, and high-throughput sequencing technology was employed. The aim was to examine the differences in the regulation of potato rhizosphere fungal community structure and diversity by different organic materials under the same experimental conditions, with the goal of identifying superior soil amelioration practices for this region, constructing a microbiota more conducive to crop growth, and providing a theoretical basis for sustainable fertilization strategies in local potato production. We hypothesized that: (1) compared with chemical fertilizer alone, the combined application of sheep manure, biochar, or maize straw with chemical fertilizer would significantly increase soil nutrient contents and crop yield, but the magnitude of these effects would be material-specific; (2) the application of different organic materials combined with chemical fertilizer would significantly increase the alpha diversity and alter the beta diversity of the potato rhizosphere fungal community; and (3) different fungal genera would respond differently to organic materials, with soil organic matter and total nitrogen being the key environmental factors driving fungal community variation.

## 2 Materials and methods

### 2.1 Overview of the pilot zone

This study was conducted at an experimental plot in Fuqiang Village, Wushengyi Town, Yongdeng County, Lanzhou City, Gansu Province (36°55′45′′N, 103°08′23′′E). The study area has an average elevation of 2,300 m, an annual average temperature of approximately 4 °C, an annual average precipitation of 380–450 mm, and a frost-free period of 100–125 days. The topography of the study area belongs to the semi-arid and semi-humid Eryin Mountain region, and the soil type is chestnut calcareous soil.

### 2.2 Experimental design

The experiment was conducted from April to October 2025. Four treatments were established: chemical fertilizer alone as the control (CF), sheep manure combined with chemical fertilizer (T1), biochar combined with chemical fertilizer (T2), and maize straw combined with chemical fertilizer (T3). The application rates of chemical fertilizers were N 180 kg⋅hm^–2^, P_2_O_5_ 120 kg⋅hm^–2^, and K_2_O 90 kg⋅hm^–2^. The organic materials were applied at the following rates: composted sheep manure at 27,000 kg⋅hm^–2^ (containing C 28.1%, N 0.71%, P_2_O_5_ 0.6%, and K_2_O 0.64%), biochar at 15,000 kg⋅hm^–2^ (containing C 49.7%, N 1.1%, P_2_O_5_ 0.23%, and K_2_O 0.75%), and maize straw at 5,250 kg⋅hm^–2^ (containing C 44%, N 0.8%, P_2_O_5_ 0.36%, and K_2_O 1.05%). All organic materials were applied in addition to the chemical fertilizer rates used in the CF treatment. All fertilizers and organic materials were applied as a single basal dressing before sowing and incorporated into the soil by rotary tillage. The maize straw was chopped into 3–5 cm segments prior to application, and the composted sheep manure had a moisture content below 30%. The test potato variety was “Wotu 5,” a cultivar commonly used by local farmers.

The experiment was arranged in a randomized complete block design (RCBD) with three replications per treatment, totaling 12 plots. Each plot covered an area of 30 m^2^, and treatments were randomly assigned within each block. Potatoes were cultivated in ridges with full plastic film mulching. The ridge width was 60 cm, and the ridge height was 15 cm. Seed tubers were planted on both sides of the ridges at a spacing of 35–40 cm. Field management practices during the growing season, including weeding and irrigation, followed local conventional procedures.

### 2.3 Sample collection

At potato harvest in October 2025, rhizosphere soil samples were collected from each plot using a five-point sampling method, with five plants randomly selected per plot. Each potato plant was carefully uprooted together with the soil adhering to its roots, and the loose soil was removed by gentle shaking. A sterile soft-bristled brush was used to brush unidirectionally along the root growth direction, and the rhizosphere soil was directly collected into sterile self-sealing bags placed beneath the roots. After thorough mixing, the soil was divided into two aliquots, each approximately 500 g, and placed into sterile self-sealing bags. One aliquot was kept in an ice box (4 °C) and transported to the laboratory on the same day, where it was stored at −80 °C for high-throughput sequencing. The other aliquot was air-dried in a cool and ventilated place for the determination of soil physicochemical properties. Afterward, all tubers from each plot were manually harvested and weighed to record the fresh tuber yield, which was then converted to yield per hectare.

### 2.4 Determination of soil chemical properties

Following the methods for determining soil physicochemical properties described in Soil Agrochemical Analysis (Bao, 2000), pH was measured using a pH meter (PB-10, Sartorius, Göttingen, Germany) to measure pH (soil-to-water ratio of 5:1), the K_2_Cr_2_O_7_ oxidation-external heating method to determine soil organic matter (SOM), the semi-micro open method to determine total nitrogen (TN), and the NaOH fusion-molybdenum-antimony colorimetric method to determine total phosphorus (TP), NaOH fusion-flame photometric method was used to determine total potassium (TK); the alkaline hydrolysis diffusion method was used to determine alkaline hydrolysis nitrogen (AN); the NaOH extraction-molybdenum-antimony colorimetric method was used to determine available phosphorus (AP); and the NH_4_C_2_H_3_O_2_ extraction-flame photometric method was used to determine available potassium (AK).

### 2.5 Determination of soil fungal community structure and diversity

Total microbial community DNA was extracted using the FastPure Soil DNA Isolation Kit (Magnetic Bead) (MJYH, Shanghai, China). DNA extraction quality was assessed via 1% agarose gel electrophoresis, and DNA concentration was measured using the NanoDrop2000 (Thermo Scientific, USA). Using the extracted DNA as a template, PCR amplification of the ITS gene was performed with barcoded primers ITS1F (5′-CTTGGTCATTTAGAGGAAGTAA-3′) and ITS2R (5′-GCTGCGTTCTTCATCGATGC-3′) (Liu C. S. et al., 2016). The PCR reaction system consisted of: 10 μL 2× Pro Taq Ready-to-Use Buffer, 0.8 μL upstream primer (5 μM), 0.8 μL downstream primer (5 μM), template DNA (10 ng/μL) made up to 20 μL. The amplification program was as follows: (1) 95 °C pre-denaturation for 3 min; (2) 35 cycles (denaturation at 95 °C for 30 s, annealing at 55 °C for 30 s, extension at 72 °C for 45 s); (3) Extension at 72 °C for 10 min; followed by storage at 10 °C (PCR instrument: ABI GeneAmp 9700, Thermo Fisher Scientific, USA). PCR products were recovered using a 2% agarose gel and purified with a DNA gel purification kit (PCR Clean-Up Kit, China Yuhua). The purified products were quantified using Qubit 4.0 (Thermo Fisher Scientific, USA).

Library construction was performed using the NEXTFLEX Rapid DNA-Seq Kit (Illumina, San Diego, CA, USA) according to the manufacturer’s instructions. The procedure included: (1) adapter ligation; (2) magnetic bead-based selection to remove adapter-dimers; (3) PCR amplification to enrich the library templates; and (4) magnetic bead recovery of the PCR products to obtain the final library. Sequencing was conducted on the Illumina Nextseq 2000 platform (Shanghai Majorbio Bio-pharm Technology Co., Ltd., Shanghai, China). The raw sequence data have been deposited in the NCBI SRA database under accession number PRJNA1473284.

### 2.6 Data processing

Raw FASTQ files were de-multiplexed using an in-house perl script, and then quality-filtered by fastp (Chen et al., 2018) (version 0.23.4)^1^ and merged by FLASH (Magoč and Salzberg, 2011) (version 1.2.11)^2^ with the following criteria: (1) the reads were truncated at any site receiving an average quality score of <20 over a 50 bp sliding window, and the truncated reads shorter than 50 bp were discarded, reads containing ambiguous characters were also discarded; (2) only overlapping sequences longer than 10 bp were assembled according to their overlapped sequence. The maximum mismatch ratio of the overlap region is 0.2. Reads that could not be assembled were discarded; (3) Samples were distinguished according to the barcode and primers, and the sequence direction was adjusted, exact barcode matching, 2 nucleotide mismatch in primer matching. Then the optimized sequences were clustered into operational taxonomic units (OTUs) using USEARCH (Edgar, 2013; Stackebrandt and Goebel, 1994) (version 11)^3^ with 97% sequence similarity level.

The most abundant sequence for each OTU was selected as a representative sequence. To minimize the impact of sequencing depth on subsequent data analysis, the abundance data from the 12 samples were smoothed. OTU taxonomic annotation was performed using the RDP Classifier (Wang, 2007) (version 2.11)^4^ against the UNITE 8.0 (ITS_fungi) gene database (confidence threshold set at 70%).

### 2.7 Statistical analysis

Soil physicochemical properties and crop yield data were analyzed using SPSS 27.0 and Excel 2024 software. One-way analysis of variance (ANOVA) followed by Tukey’s HSD *post-hoc* test was used to compare these parameters among treatments. Microbial data analyses were performed on the Majorbio Cloud Platform^5^. Alpha diversity indices were calculated using mothur (Schloss et al., 2009)^6^, and differences in Alpha diversity among treatments were assessed using one-way ANOVA, followed by Tukey–Kramer *post-hoc* tests. Differences in the relative abundances of fungal taxa among treatments were evaluated using one-way ANOVA, with *post hoc* multiple comparisons conducted using LSD tests. Beta diversity was assessed using principal coordinate analysis (PCoA) based on Bray–Curtis distances, combined with ANOSIM and Adonis (PERMANOVA) to evaluate overall differences in fungal community structure among treatments. LEfSe (Linear Discriminant Analysis Effect Size) (Segata et al., 2011)^7^ was performed to identify fungal taxa with significantly different abundances among groups from phylum to genus level (LDA > 3.8, *p* < 0.05). The analysis procedure involved the Kruskal–Wallis rank-sum test for initial screening of differentially abundant taxa, followed by unpaired Wilcoxon rank-sum tests to verify the consistency of pairwise comparisons, and linear discriminant analysis (LDA) to estimate the effect size of each taxon. Redundancy analysis (RDA) and variation partitioning analysis (VPA) were performed using the vegan package in R software (version 3.3.1) to elucidate the effects of soil physicochemical properties on fungal community structure and to quantify their relative contributions.

## 3 Results and analysis

### 3.1 Analysis of rhizosphere soil nutrient contents and potato yield

The nutrient contents of potato rhizosphere soil and crop yield under different treatments are shown in Table 1. Compared with CF, treatments T1, T2, and T3 significantly increased soil organic matter content (*p* < 0.05), with T2 having the highest content, representing an increase of 86.85% over CF. Compared with CF, treatments T1, T2, and T3 also significantly increased soil total nitrogen content (*p* < 0.05), with T2 having the highest content, an increase of 84.29% over CF. Significant differences in alkaline hydrolyzable nitrogen content were observed between T2, T3, and CF (*p* < 0.05), with T2 and T3 increasing by 35.59% and 23.74%, respectively. Compared with CF, all three treatments significantly increased soil available phosphorus content (*p* < 0.05), with T3 exhibiting the highest content, an increase of 68.18% over CF. Treatments T1 and T3 showed significant differences in available potassium content compared with CF (*p* < 0.05), with increases of 22.17% and 17.03%, respectively.

**TABLE 1:** Soil nutrient contents and crop yield in potato rhizosphere soil under different fertilization treatments.

Indicator	CF	T1	T2	T3
pH	8.36 ± 0.01a	8.38 ± 0.05a	8.36 ± 0.03a	8.36 ± 0.02a
Soil organic matter (g⋅kg^–1^)	10.57 ± 0.52c	13.54 ± 0.54b	19.75 ± 0.94a	15.60 ± 1.06b
Total nitrogen (g⋅kg^–1^)	0.70 ± 0.04c	1.02 ± 0.09b	1.29 ± 0.02a	1.06 ± 0.03b
Total phosphorus (g⋅kg^–1^)	0.79 ± 0.04a	0.79 ± 0.01a	0.79 ± 0.03a	0.80 ± 0.05a
Total potassium (g⋅kg^–1^)	22.78 ± 0.14a	22.55 ± 0.38a	23.28 ± 0.37a	22.96 ± 0.17a
Alkaline hydrolyzable nitrogen (mg⋅kg^–1^)	37.15 ± 3.37c	38.91 ± 1.17bc	50.37 ± 2.14a	45.97 ± 2.11ab
Available phosphorus (mg⋅kg^–1^)	9.49 ± 0.68b	14.91 ± 1.10a	13.65 ± 1.35a	15.96 ± 0.61a
Available potassium (mg⋅kg^–1^)	227.09 ± 3.82c	277.43 ± 12.55a	243.32 ± 6.58bc	265.76 ± 7.97ab
Yield (t⋅hm^–2^)	46.05 ± 0.55b	60.05 ± 1.25a	62.55 ± 1.08a	46.35 ± 0.51b

The significance of differences was determined by the letter marking method (*p* < 0.05). Different lowercase letters in the same column indicate statistically significant differences among treatments. CF, chemical fertilizer alone; T1, sheep manure + chemical fertilizer; T2, biochar + chemical fertilizer; T3, corn stover + chemical fertilizer.

Regarding crop yield, T1 and T2 differed significantly from CF (*p* < 0.05), with yield increases of 30.40% and 35.84%, respectively.

### 3.2 Analysis of potato rhizosphere soil fungal OTUs

ITS amplicon sequencing was performed to profile the soil fungi in the potato rhizosphere under different fertilization treatments. In this study, 12 soil samples were sequenced, yielding 881,297 valid sequences with an average length of 240 bp. To minimize the impact of sequencing depth on subsequent analysis, the 12 samples were rarefied to an even depth of 63,008 sequences per sample, yielding a total of 1,366 OTUs. Coverage curves for each treatment are shown in Figure 1. The coverage indices for all groups approached 1, indicating that the sequencing depth was sufficient to meet the accuracy requirements for subsequent experimental analysis.

**FIGURE 1:**
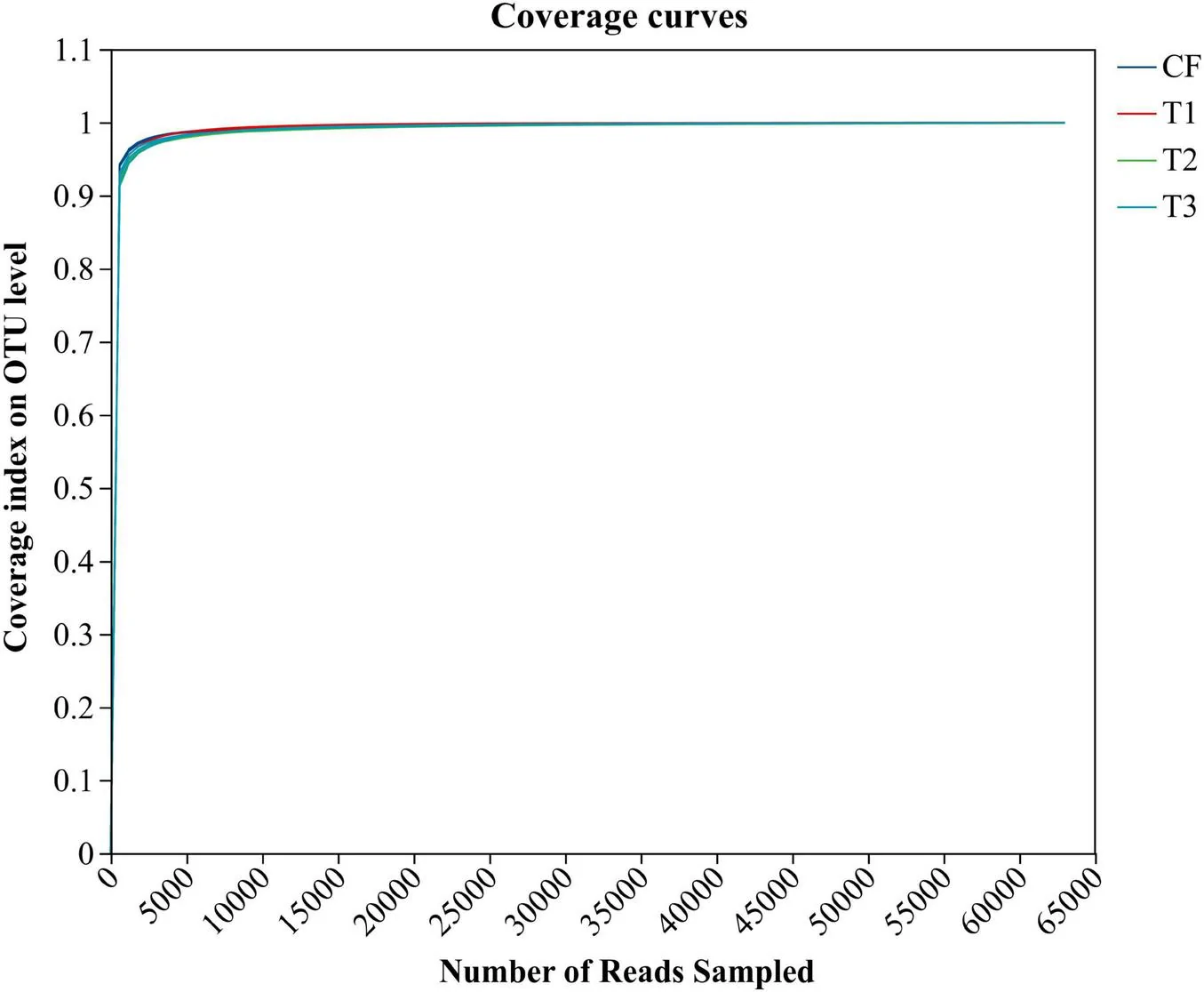
Coverage curves of fungal OTUs in potato rhizosphere soil under different fertilization treatments. CF, chemical fertilizer alone; T1, sheep manure + chemical fertilizer; T2, biochar + chemical fertilizer; T3, corn stover + chemical fertilizer.

### 3.3 Analysis of alpha diversity indices for potato rhizosphere soil fungi

As shown in Figure 2a, the Chao index followed the order: T2 > T3 > T1 > CF. Compared with CF, the fungal richness index was significantly increased by 50.14% in T2 (*p* < 0.001) and by 28.35% in T3 (*p* < 0.05). No significant difference was detected between T1 and CF (*p* > 0.05).

**FIGURE 2:**
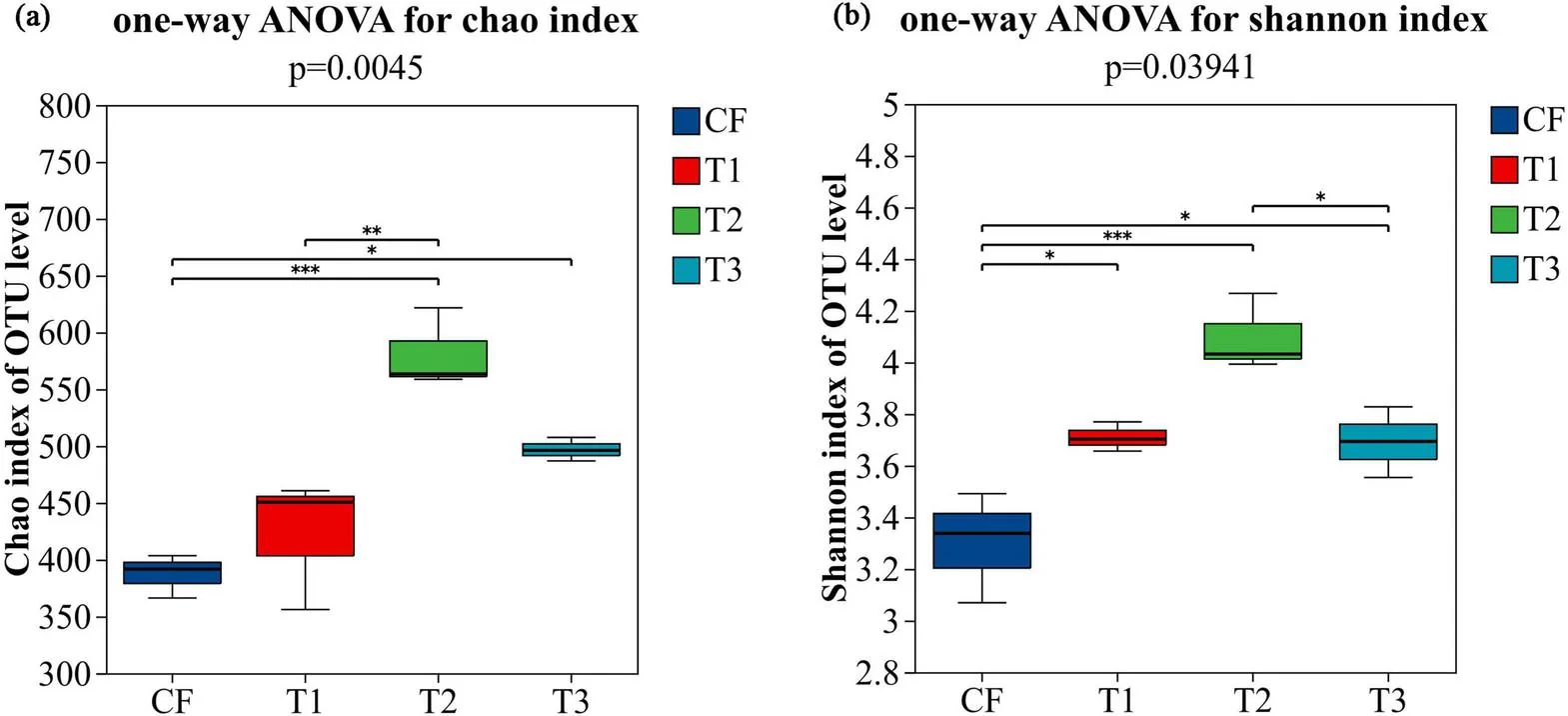
Chao index **(a)** and Shannon index **(b)** of fungal communities in potato rhizosphere soil under different fertilization treatments. CF, chemical fertilizer alone; T1, sheep manure + chemical fertilizer; T2, biochar + chemical fertilizer; T3, corn stover + chemical fertilizer. The significant levels are indicated by asterisk: *p < 0.05; **p < 0.01;***p < 0.001.

As shown in Figure 2b, the Shannon index followed the order: T2 > T1 > T3 > CF. Compared with CF, the fungal diversity index was significantly increased by 24.19% in T2 (*p* < 0.001), by 12.44% in T1 (*p* < 0.05), and by 11.89% in T3 (*p* < 0.05).

### 3.4 Analysis of fungal community composition in potato rhizosphere soil

#### 3.4.1 Phylum-level analysis of fungal communities

Community composition analysis provides a clear visualization of species differences among treatments at the same taxonomic level. As shown in Figure 3, a total of 14 phyla were detected in the fungal community at the phylum level. The top five most abundant phyla accounted for 99.53% of the total fungal community, and therefore only these dominant phyla were subjected to further analysis. Among them, Ascomycota, Mortierellomycota, Basidiomycota, and Chytridiomycota accounted for 86.12%, 6.34%, 4.24%, and 2.36% of the total fungal sequences, respectively. In addition, a small proportion of fungal sequences (approximately 2.4%) across all samples remained unclassified at the phylum level.

**FIGURE 3:**
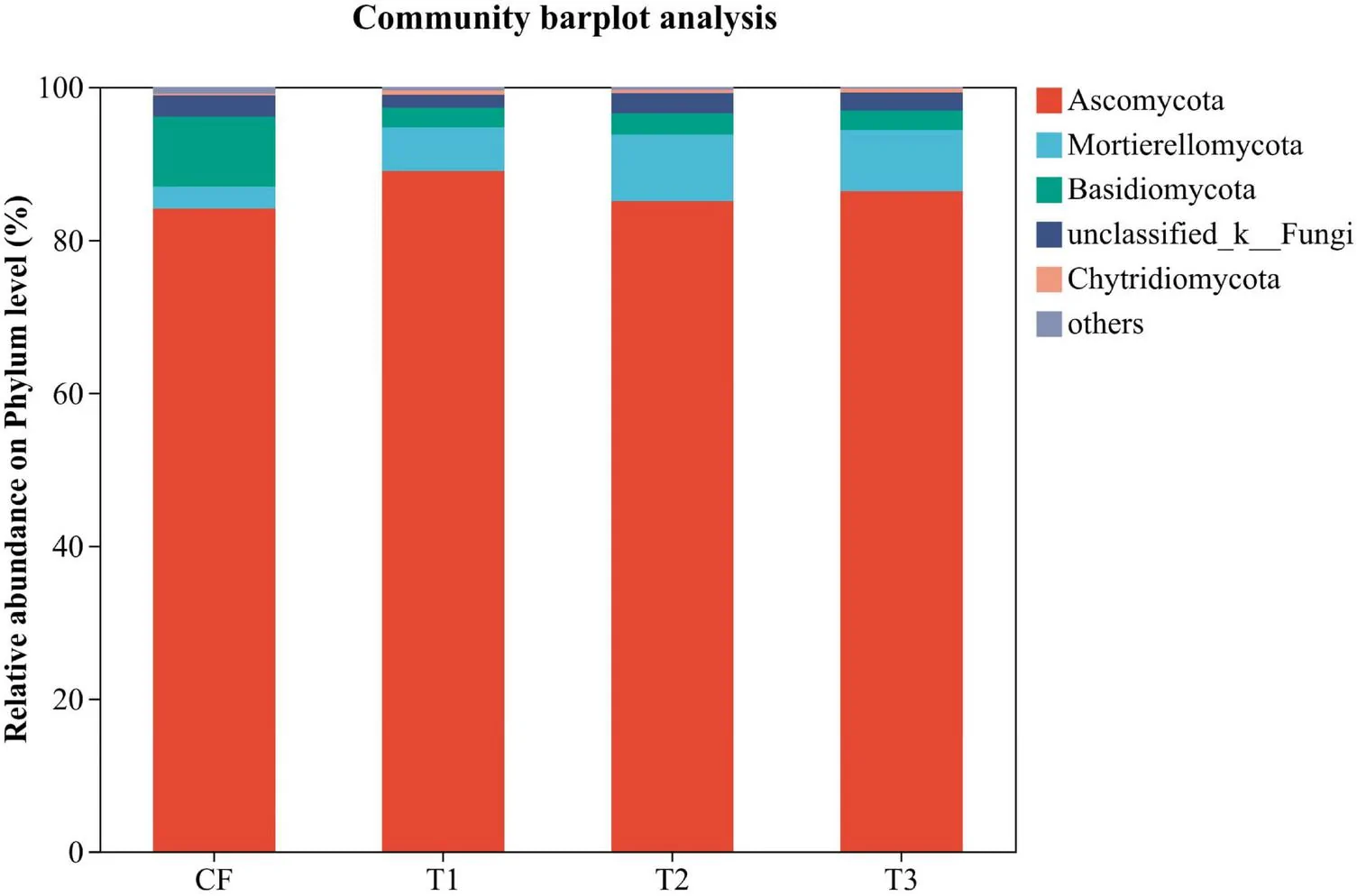
Relative abundance of fungal phyla in potato rhizosphere soil under different fertilization treatments. CF, chemical fertilizer alone; T1, sheep manure + chemical fertilizer; T2, biochar + chemical fertilizer; T3, corn stover + chemical fertilizer.

Compared with the CF treatment, the relative abundance of Mortierellomycota was significantly increased in the T2 and T3 treatments (*p* < 0.05). The relative abundance of Chytridiomycota in the T1 treatment was significantly higher than that in the CF treatment (*p* < 0.05). The relative abundance of Basidiomycota decreased in all organic amendment treatments compared with CF; however, no significant differences were detected among treatments.

#### 3.4.2 Genus-level analysis of fungal communities

A total of 392 genera were detected in the fungal community at the genus level, and the top 10 most abundant genera were selected for further analysis (Figure 4). Among them, *Plectosphaerella*, *Pseudogymnoascus*, *Alternaria*, *Mortierella*, *Scutellinia*, *Lectera*, *Pseudombrophila*, *Trichocladium*, and *Fusarium* accounted for 8.25%, 6.24%, 5.28%, 4.60%, 4.50%, 4.38%, 4.05%, 3.95%, and 3.74% of the total fungal community, respectively. In addition, approximately 9.29% of fungal sequences across all samples remained unclassified at the genus level.

**FIGURE 4:**
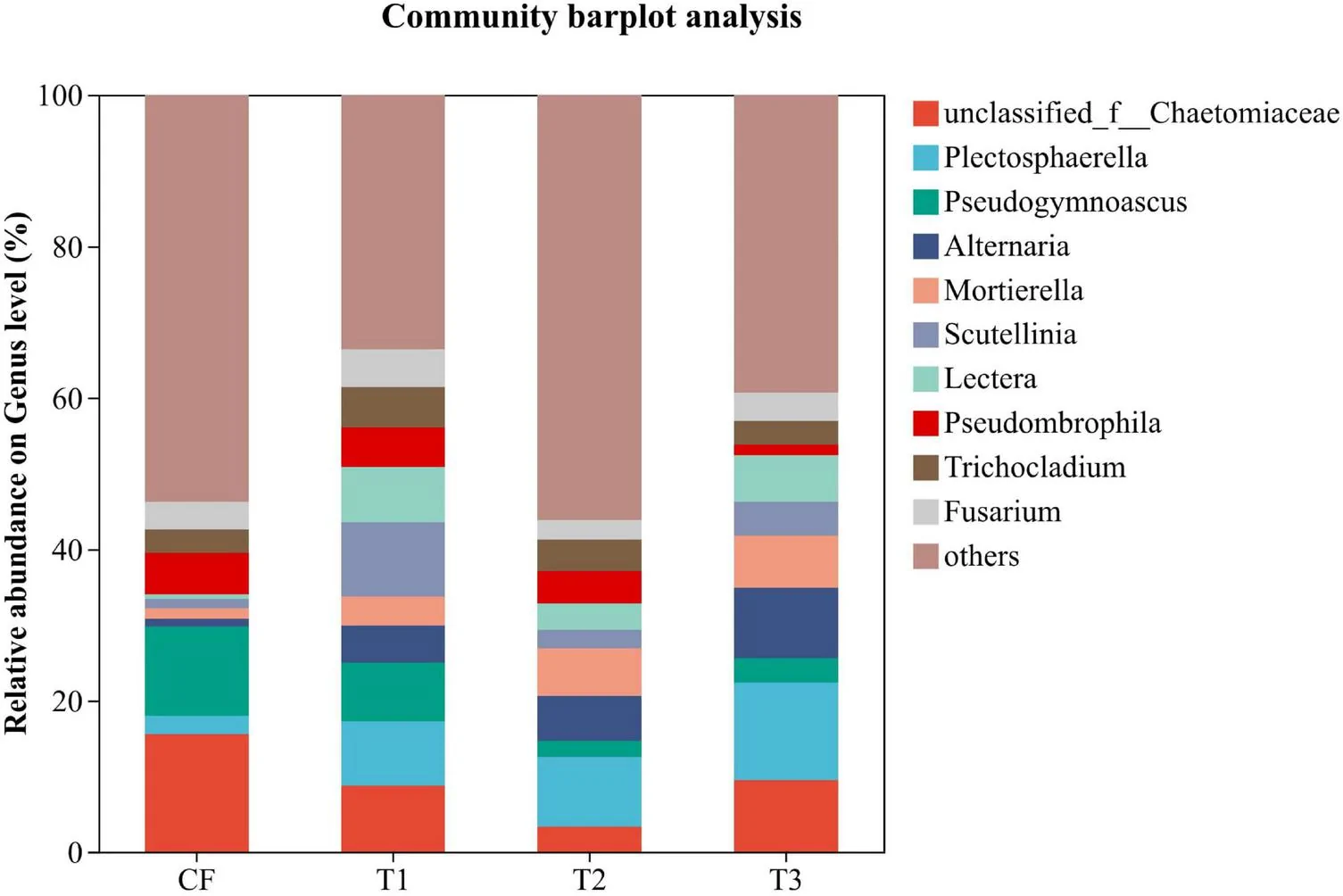
Relative abundance of fungal genera in potato rhizosphere soil under different fertilization treatments. CF, chemical fertilizer alone; T1, sheep manure + chemical fertilizer; T2, biochar + chemical fertilizer; T3, corn stover + chemical fertilizer.

Compared with CF, the relative abundance of *Plectosphaerella* was significantly increased in the T1, T2, and T3 treatments (*p* < 0.05). The relative abundance of *Pseudogymnoascus* was significantly decreased in the T2 and T3 treatments compared with CF (*p* < 0.05). The relative abundance of *Alternaria* was significantly increased in the T3 treatment compared with CF (*p* < 0.01). The relative abundance of *Mortierella* was significantly increased in the T2 treatment (*p* < 0.05) and highly significantly increased in the T3 treatment (*p* < 0.01) compared with CF. The relative abundance of *Scutellinia* was highly significantly increased in the T1 treatment compared with CF (*p* < 0.01). The relative abundance of *Pseudombrophila* was significantly decreased in the T3 treatment compared with CF (*p* < 0.001).

### 3.5 Beta diversity analysis of potato rhizosphere soil fungal communities

The PCoA results based on Bray-Curtis distances are shown in Figure 5. Fungal community samples from different fertilization treatments exhibited a clear separation trend in the ordination space. PC1 explained 31.3% of the community variation, and samples from the CF treatment clustered on the negative side of the PC1 axis, distinctly separated from the T1, T2, and T3 treatments, indicating that organic matter input drove the restructuring of fungal community structure. PC2 explained 16.36% of the community variation, further distinguishing the T2 treatment from T1 and T3, suggesting that different types of organic materials exerted divergent regulatory effects on fungal communities. PERMANOVA confirmed the highly significant differentiation among groups (R^2^ = 0.493, *p* = 0.001), and ANOSIM yielded a consistent result (*R* = 0.5617, *p* = 0.001).

**FIGURE 5:**
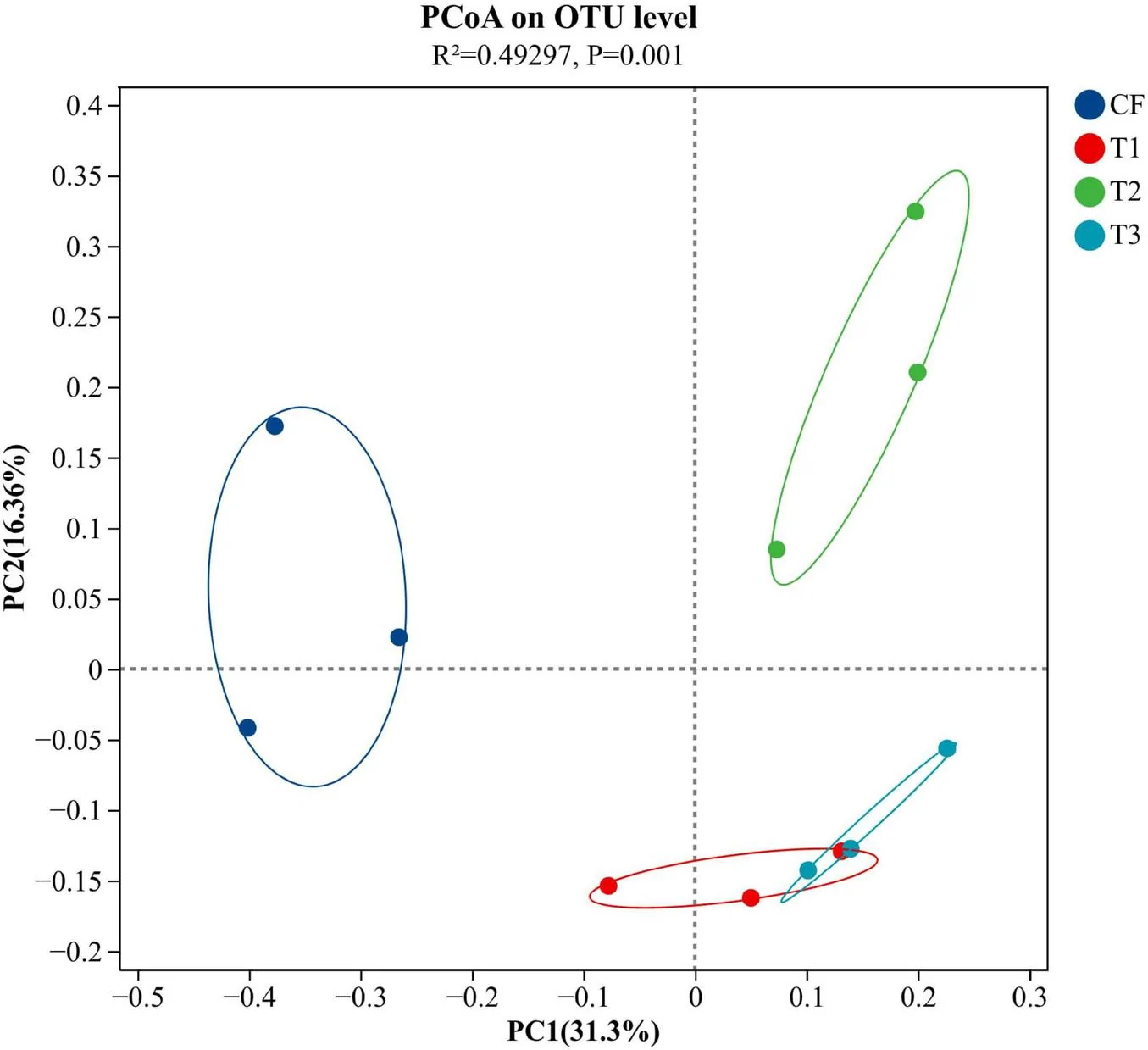
Principal coordinate analysis (PCoA) analysis of fungal community structure in potato rhizosphere soil under different treatments based on Bray-Curtis distance at the OTU level. CF, chemical fertilizer alone; T1, sheep manure + chemical fertilizer; T2, biochar + chemical fertilizer; T3, corn stover + chemical fertilizer.

### 3.6 Differential analysis of potato rhizosphere soil fungal communities

LEfSe (Linear Discriminant Analysis Effect Size) analysis was performed to reveal the differential shaping of soil microbial communities by different treatments (Figure 6). A total of seven differentially abundant fungal taxa were identified in the CF treatment, mainly including *Stachybotrys*, *Chaetomidium*, and others. Three differentially abundant fungal taxa were identified in the T1 treatment, mainly including *Scutellinia*, *s_Scutellinia_sp*, and others. Eight differentially abundant fungal taxa were detected in the T2 treatment, mainly including *Plectosphaerellaceae*, *Eurotiomycetes*, Onygenales, *Gymnoascus*, and others. Four differentially abundant fungal taxa were detected in the T3 treatment, mainly including *Mortierella*, *Furcasterigmium*, Glomerellales, and others. Among the four treatments, the T2 treatment screened out the highest number of differentially abundant fungal taxa (8 species), while the T1 treatment screened out the lowest number (3 species).

**FIGURE 6:**
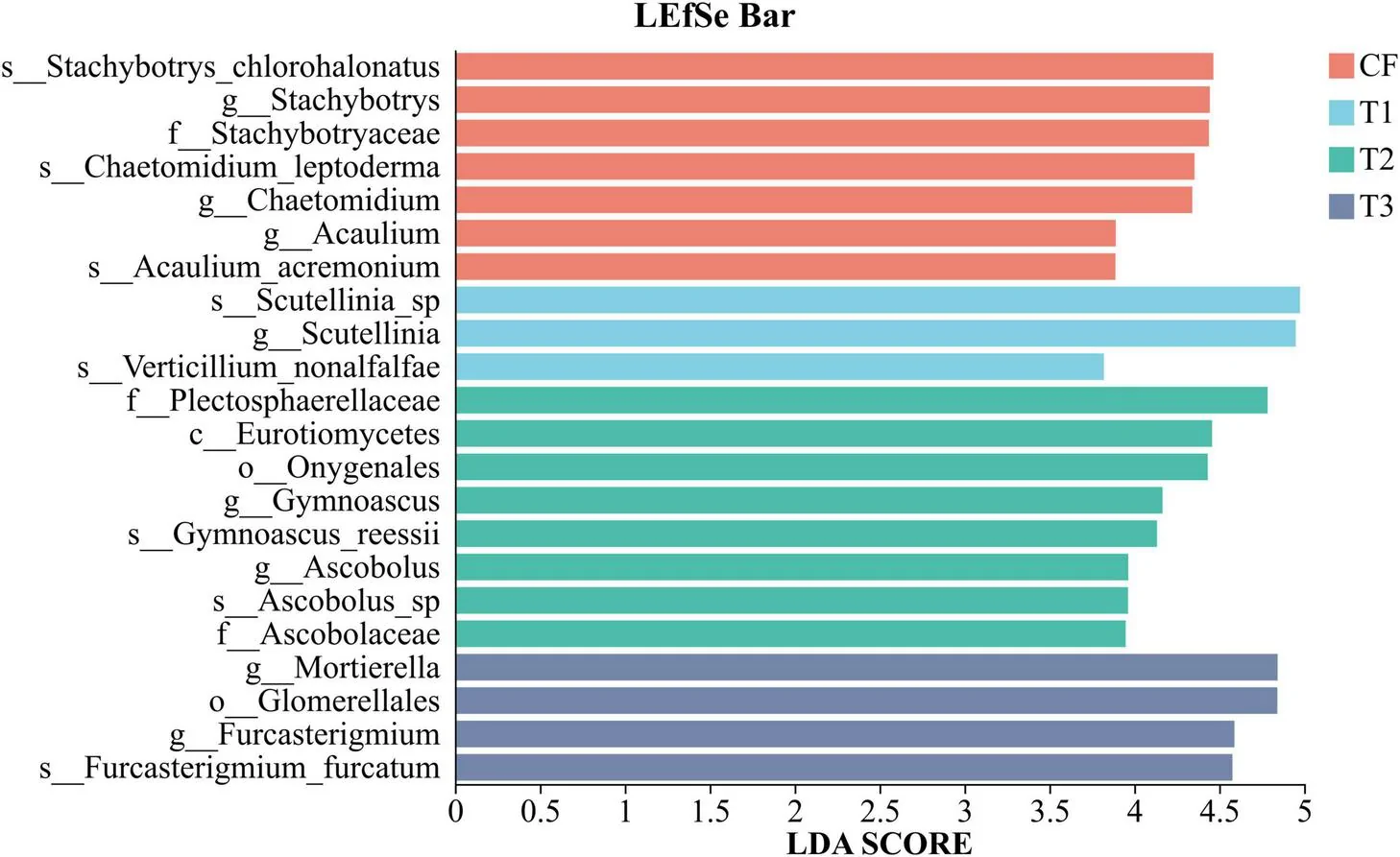
Linear Discriminant Analysis Effect Size (LEfSe) analysis of fungal communities in potato rhizosphere soil under different fertilization treatments (LDA > 3.8, *p* < 0.05). CF, chemical fertilizer alone; T1, sheep manure + chemical fertilizer; T2, biochar + chemical fertilizer; T3, corn stover + chemical fertilizer.

### 3.7 Correlation analysis between potato rhizosphere soil fungal communities and soil environmental factors

To further elucidate the main environmental factors driving soil fungal community composition, correlation analyses between fungal community composition at the genus level and soil environmental factors were performed across different treatments, and a correlation heatmap and redundancy analysis (RDA) were constructed. As shown in Figure 7, *Pseudombrophila* exhibited a significant negative correlation with available phosphorus (AP) (*p* < 0.05). Lectera showed a highly significant positive correlation with available potassium (AK) (*p* < 0.01). *Scutellinia* displayed a significant positive correlation with AK (*p* < 0.05). *Mortierella* was significantly positively correlated with soil organic matter (SOM), total nitrogen (TN), and AP (*p* < 0.05), and highly significantly positively correlated with alkaline hydrolyzable nitrogen (AN) (*p* < 0.01). *Alternaria* was significantly positively correlated with SOM and AP (*p* < 0.05). *Pseudogymnoascus* exhibited a highly significant negative correlation with SOM (*p* < 0.01) and a significant negative correlation with AN (*p* < 0.05). *Plectosphaerella* was significantly positively correlated with SOM, AP, and AN (*p* < 0.05).

**FIGURE 7:**
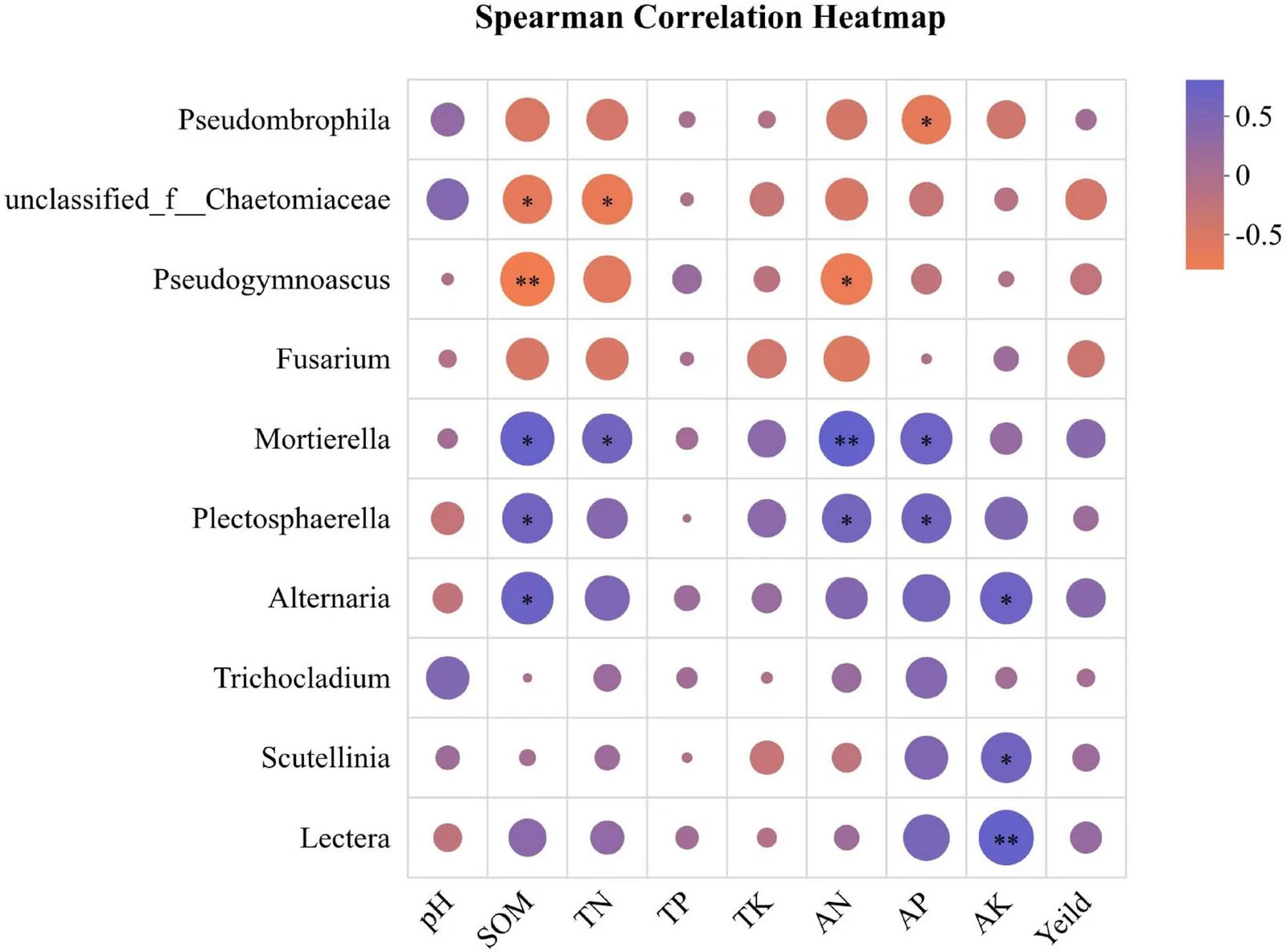
Spearman correlation heatmap between fungal genera and soil physicochemical properties, as well as crop yield, in potato rhizosphere soil under different fertilization treatments. SOM, Soil organic matter; TN, total nitrogen; TP, total phosphorus; TK, total potassium; AN, alkaline hydrolyzable nitrogen; AP, available phosphorus; AK, available potassium. CF, chemical fertilizer alone; T1, sheep manure + chemical fertilizer; T2, biochar + chemical fertilizer; T3, corn stover + chemical fertilizer. The significant levels are indicated by asterisk: *p < 0.05; **p < 0.01.

The redundancy analysis (RDA) results are presented in Figure 8. Total nitrogen (TN) and available phosphorus (AP) showed positive correlations with crop yield. TN was identified as the most important environmental driver of fungal community variation (*p* < 0.05).

**FIGURE 8:**
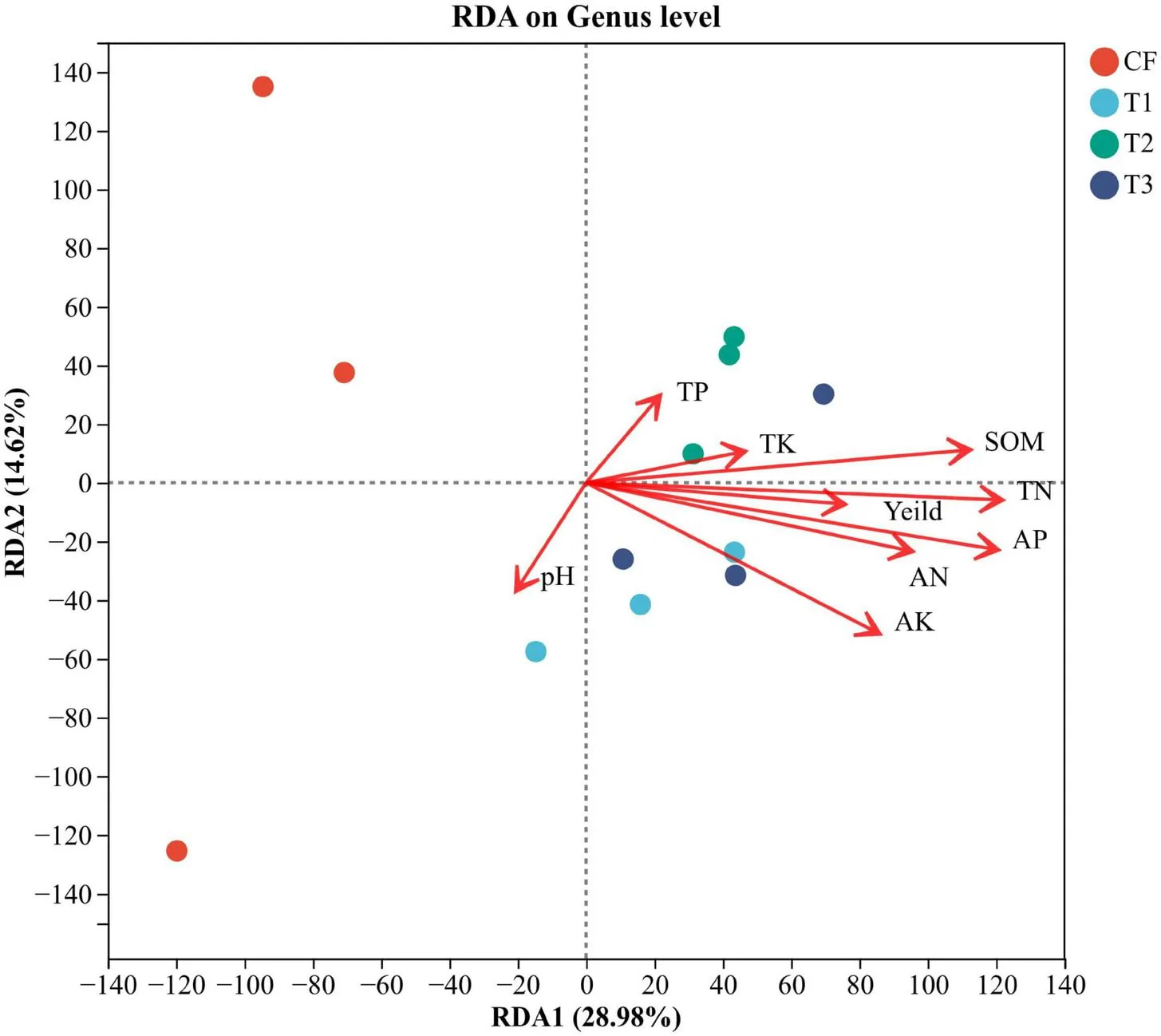
Redundancy analysis (RDA) of fungal community at genus level and soil environmental factors in potato rhizosphere soil. SOM, Soil organic matter; TN, total nitrogen; TP, total phosphorus; TK, total potassium; AN, alkaline hydrolyzable nitrogen; AP, available phosphorus; AK, available potassium. CF, chemical fertilizer alone; T1, sheep manure + chemical fertilizer; T2, biochar + chemical fertilizer; T3, corn stover + chemical fertilizer.

## 4 Discussion

The combined application of organic materials and chemical fertilizers is a major strategy for improving soil quality and crop yield. In this study, the T1, T2, and T3 treatments all significantly increased soil organic matter content, with the T2 treatment showing the most pronounced effect. This may be because biochar itself contains a large amount of organic matter and is rich in highly aromatic stable carbon. Due to its chemical stability, biochar is not easily decomposed and mineralized by microorganisms after being applied to soil, and can persist as a stable organic carbon fraction, thereby contributing to the effective accumulation of organic matter (Chu et al., 2025). Corn stover and sheep manure also contain substantial amounts of organic carbon components, and most studies have shown that their application can increase soil organic matter content (He Z. M. et al., 2025; Li et al., 2018). However, compared with biochar, the organic matter in corn stover and sheep manure is more readily decomposed by microorganisms (Li et al., 2018), leading to a faster but less persistent increase in soil organic matter. Regarding soil nitrogen, T2 showed the greatest improvement in total nitrogen and alkaline hydrolyzable nitrogen contents. On the one hand, biochar itself contains considerable nitrogen, and the interaction between biochar and nitrogen fertilizer can stimulate the activity of nitrogen-cycling enzymes in the soil, providing a suitable habitat for nitrogen-transforming microorganisms, thereby promoting nitrogen retention and transformation in the soil (Ibrahim et al., 2020). On the other hand, biochar has a highly developed porous structure and a large specific surface area. After being applied to soil, it can effectively retain ammonium and nitrate nitrogen in soil solution through physical adsorption and ion exchange, reducing nitrogen losses due to leaching and volatilization (Kohira et al., 2025). The T3 treatment also significantly increased the soil total nitrogen and alkaline hydrolyzable nitrogen contents. This was primarily because corn stover, as an organic material with a high carbon-to-nitrogen ratio, stimulated soil microbial activity upon application; during the decomposition of the stover, microorganisms accelerated the mineralization and release of organic nitrogen in the soil (Hu et al., 2015). The T1 treatment significantly increased total nitrogen content, but the increase in alkaline hydrolyzable nitrogen was not significant. Although the application rate of well-decomposed sheep manure was higher than that of corn stover and its carbon-to-nitrogen ratio was lower, the nitrogen in sheep manure consists primarily of complex organic nitrogen, which requires a longer time to mineralize and release. It may not have been fully converted into readily available nitrogen for crop uptake within a single growing season (Song et al., 2025). In terms of soil phosphorus, the T3 treatment exhibited the most prominent increase in available phosphorus content. This is because corn stover decomposes under microbial action, releasing organic acids that can dissolve insoluble phosphate minerals in the soil, thereby activating and releasing fixed phosphorus into the soil solution (Fan, 2007). The T1 and T2 treatments also significantly increased soil available phosphorus content, mainly due to the fact that sheep manure itself contains certain amounts of inorganic phosphorus and easily mineralizable organic phosphorus, which can directly supplement the available phosphorus pool after application (Liu et al., 2021). Other studies have confirmed that biochar application can increase both inorganic and organic phosphorus content in soil, while significantly reducing the soil’s adsorption capacity for phosphate ions, thereby decreasing phosphorus fixation (Zhang et al., 2026). The T1 treatment showed the most significant increase in available potassium content. Potassium in sheep manure exists mainly in ionic form with strong water solubility, allowing rapid release into the soil solution after application, directly supplementing the available potassium pool (Hou et al., 2021). The T3 treatment also significantly increased available potassium content. Studies have found that straw return can promote the mineralization of organic potassium, significantly increasing soil available potassium content (Tan et al., 2009). Although the combined application of biochar increased available potassium content to some extent, the increase was not significant. This may be because biochar has a limited direct supplemental effect on potassium, with its main role being an indirect influence on potassium availability through the improvement of soil physicochemical properties (He W. et al., 2025).

Soil fungi play crucial roles in soil humus formation, nutrient cycling, organic matter stabilization, and suppression of plant pathogens, making them one of the key indicators for evaluating soil fertility and health (De Corato, 2020). In this study, the Chao and Shannon indices were employed to assess the species richness and diversity of soil fungal communities under different fertilization treatments. The results showed that, compared with chemical fertilizer alone, the T1, T2, and T3 treatments all increased soil fungal richness and diversity, with the T2 treatment exhibiting the most pronounced effect. This may be attributed to two main factors. On the one hand, organic materials themselves contain various mineral nutrients required for microbial life activities (Bai et al., 2025; Luo et al., 2015). On the other hand, the majority of fungi are strictly aerobic microorganisms (Deng et al., 2022); the combined application of biochar and maize straw can improve soil porosity and aggregate structure, thereby facilitating fungal growth and reproduction. Consistent with our findings, Chang et al. (2024) and Ren et al. (2021) demonstrated that biochar application enhanced soil fungal community richness and diversity. Similarly, Gao et al. (2023) found that chemical fertilizer combined with sheep manure significantly increased soil microbial richness and diversity. Zhang et al. (2021) also reported that straw return combined with conventional fertilization significantly improved fungal richness and diversity. Collectively, these studies are in agreement with the results of the present experiment.

Analysis of the soil fungal community composition at the phylum level revealed that both T2 and T3 treatments significantly increased the abundance of Mortierellomycota. Mortierellomycota is a typical soil saprotrophic fungus that plays an important role in soil material cycling (Wang et al., 2018). It can not only promote plant growth but also specifically inhibit certain pathogens, thereby controlling corresponding soilborne diseases (Eroshin and Dedyukhina, 2002). The combined application of biochar, maize straw, and chemical fertilizers supplemented soil carbon and nitrogen sources, which favored the growth of saprotrophic fungi. This is consistent with the findings of Li et al. (2025). The T1 treatment significantly increased the abundance of Chytridiomycota in the soil. Most Chytridiomycota are decomposers in the soil; the application of sheep manure increased soil organic matter content, leading to an increase in Chytridiomycota abundance (Huo and Yu, 2018; Yelle et al., 2009), which is consistent with the results of Zhao (2021). At the fungal genus level, the T1, T2, and T3 treatments all significantly increased the abundance of *Plectosphaerella*. *Plectosphaerella* can cause root rot and wilt diseases in various crops, but some strains also have the ability to mineralize organic nitrogen sources and participate in soil organic matter decomposition (Chen, 2025). In this study, the abundance of *Plectosphaerella* was significantly positively correlated with available phosphorus and organic matter content. The combined application of organic materials increased soil available phosphorus and organic matter content, thereby increasing the abundance of *Plectosphaerella*. Both T2 and T3 significantly reduced the abundance of *Pseudogymnoascus*. *Pseudogymnoascus* belongs to the phylum Ascomycota. The decrease in its abundance may be related to competition for ecological niches; the increased abundance of fungi such as *Mortierella* may have compressed the living space of *Pseudogymnoascus*.

The T3 treatment significantly increased the abundance of *Alternaria*. Some *Alternaria* species are decomposers in soil (Kang et al., 2013), and the combined application of organic materials provided optimal nutrient substrates for these efficient saprotrophic decomposers, while also providing favorable conditions for their proliferation (Gong et al., 2019), resulting in an increase in *Alternaria* abundance. Previous studies have also found that the combined application of organic materials significantly increased the relative abundance of *Alternaria* (Li, 2022; Yan et al., 2018). Some *Alternaria* species are parasitic fungi that can cause diseases in various plants, especially crops and economic plants. Therefore, disease monitoring in potato production should be strengthened in future long-term experiments. The abundances of *Mortierella* was significantly increased in both the T2 and T3 treatments. *Mortierella* belongs to the phylum Mortierellomycota and is a typical soil saprotroph. *Mortierella* showed a significant positive correlation with organic matter and total nitrogen. The increase in soil organic matter provided a nutrient source for soil saprotrophs, contributing to the increased abundance of *Mortierella*. This further confirms that the abundance of Mortierellomycota is associated with the increase in soil nutrients following the return of organic materials. The T1 treatment significantly increased the abundance of *Scutellinia*. *Scutellinia* is mostly terrestrial or saprotrophic on decaying wood (Bi et al., 1990), capable of decomposing complex organic compounds such as cellulose and lignin in the soil, converting them into nutrients that can be absorbed by plants, thereby promoting nutrient cycling. Sheep manure is rich in organic matter, cellulose, and hemicellulose, which provided a high-quality carbon source for *Scutellinia* (Yang Z. Y. et al., 2024), leading to an increase in its abundance. The T3 treatment significantly reduced the abundance of *Pseudombrophila*, which belongs to the phylum Ascomycota. Current research on *Pseudombrophila* is limited, and its characteristics and ecological role in soil require further investigation.

The combined application of different organic materials exerted significant regulatory effects on soil fungal community Beta diversity. Principal coordinate analysis (PCoA) revealed that samples from the T1 treatment clustered on the negative side of the PC1 axis, showing a highly significant separation from the other three treatments. Notably, the soil fungal communities in the T1 and T3 treatments exhibited a certain degree of similarity. This may be attributed to the fact that both maize straw and sheep manure introduced organic compounds such as cellulose and hemicellulose into the soil (Ji et al., 2022; Yang Z. Y. et al., 2024), which likely drove similar successional trajectories of the fungal communities.

Linear Discriminant Analysis Effect Size analysis revealed that the T2 treatment harbored the highest number of differentially abundant fungal taxa, mainly comprising saprotrophic fungi such as *Gymnoascus*, Eurotiomycetes, and *Ascobolus*, which are conducive to the decomposition of soil organic matter and nutrient cycling. The T1 treatment exhibited the fewest differentially abundant taxa, primarily including *Scutellinia* and *Verticillium nonalfalfae*. *Verticillium nonalfalfae* is a facultative plant pathogenic fungus within the *Verticillium* (Pedersen et al., 2025). Its enrichment in the T1 treatment suggests that sheep manure combined with chemical fertilizer may have created a favorable microhabitat for certain facultative pathogens. The T3 treatment was characterized by differentially abundant taxa including *Mortierella* and *Furcasterigmium*, indicating that straw return stimulated the proliferation of specific cellulose-decomposing fungi (Zheng et al., 2025). Collectively, these results demonstrate that different organic materials promoted the enrichment of distinct fungal populations. Correlation analysis between soil fungal communities and environmental factors revealed that total nitrogen (TN), available phosphorus (AP), soil organic matter (SOM), and alkaline hydrolyzable nitrogen (AN) were the main environmental factors shaping the rhizosphere fungal community of potato, among which TN was identified as the most important environmental driver of fungal community variation. This finding is consistent with the results of Xie et al. (2025).

## 5 Conclusion

Based on the single-season analysis of fungal community structure and diversity in potato rhizosphere soil, the following conclusions were drawn: (1) Compared with chemical fertilizer alone (CF), the combined application of different organic materials with chemical fertilizer improved soil nutrient status and crop yield. Among them, the T1 treatment exhibited the best performance in increasing available potassium (AK), the T2 treatment showed the greatest improvement in soil organic matter (SOM), total nitrogen (TN), and crop yield, and the T3 treatment was most effective in enhancing available phosphorus (AP). (2) The Alpha diversity of fungal communities exhibited differential single-season responses to the various fertilization measures. Both T2 and T3 treatments significantly increased the Chao index, and T1, T2, and T3 treatments all significantly increased the Shannon index, with T2 showing the greatest improvement. All organic material combined treatments resulted in a significant shift in Beta diversity away from the CF treatment, indicating pronounced community restructuring. (3) At the genus level, the T3 treatment induced an increase in the abundances of *Alternaria* and *Mortierella*, along with a decrease in *Pseudogymnoascus* abundance; the T2 treatment also significantly increased the abundance of *Mortierella*. Soil total nitrogen was identified as the dominant environmental driver regulating fungal community variation. In summary, under the single-season conditions of this experiment, the T2 treatment exhibited the most pronounced effects in enhancing soil fungal diversity and improving crop yield; however, its long-term ecological effects and potential risks of plant pathogens require further clarification through multi-year field trials.

## 6 Limitations and future perspectives

This study, based on a single-season potato field experiment conducted from April to October 2025, revealed the short-term effects of combined application of different organic materials with chemical fertilizer on rhizosphere soil fungal communities. Due to the pronounced temporal dependence of microbial community responses to organic amendments, the single-season observations primarily reflected transient community reassembly driven by initial substrate availability during the first growing season after fertilization, and thus could not predict the successional direction or stable state of community structure under multi-year continuous application. Yan et al. (2018) found through a 3-years field experiment that biochar addition increased the relative abundance of *Alternaria* fungi, which is consistent with the results of the present study. In contrast, Zhu et al. (2024), based on a 21-years long-term field experiment, found that combined application of chemical fertilizer and organic manure reduced the abundance of potential pathogenic fungi such as *Alternaria alternata* and *Gibberella avenacea* in soil. These findings indicate that the effects of organic amendments on fungal communities exhibit pronounced temporal accumulation and directional uncertainty, and that short-term experimental results cannot be directly extrapolated to multi-year scales.

Therefore, future research should conduct 3–5 years of fixed-site field trials to track the inter-annual successional trajectories of fungal communities under different fertilization treatments, with particular attention to the dynamic changes in the relative abundances of dominant taxa and potential pathogenic taxa (e.g., *Alternaria* and *Verticillium nonalfalfae*), in order to determine whether the community structure tends toward a new stable state.

## Data Availability

The datasets presented in this study can be found in online repositories. The names of the repository/repositories and accession number(s) can be found in the article/Supplementary material.

## Footnotes

1
https://github.com/OpenGene/fastp

2
https://ccb.jhu.edu/software/FLASH/index.shtml

3
http://drive5.com/usearch/

4
https://sourceforge.net/projects/rdp-classifier/

5
https://cloud.majorbio.com

6
http://www.mothur.org/wiki/Calculators

7
http://huttenhower.sph.harvard.edu/LEfSe
