# Supplementary material for: Single-season responses of rhizosphere fungal community structure and diversity to combined application of different organic materials in potato
Source: Front Microbiol. 2026 Sep 24;17:1853956. doi: 10.3389/fmicb.2026.1853956 (PMC13645750; doi:10.3389/fmicb.2026.1853956)
Supplement: Supplementary file 1 [file Data_Sheet_1.docx]

Supplementary Material

# 1 Supplementary Figures and Tables

## 1.1 Supplementary Tables

**Supplementary Table 1.** Soil nutrient contents and crop yield in potato rhizosphere soil under different fertilization treatments.

|  | CF | T1 | T2 | T3 |
| --- | --- | --- | --- | --- |
| pH | 8.36±0.01a | 8.38±0.05a | 8.36±0.03a | 8.36±0.02a |
| Soil Organic matter (g·kg⁻¹) | 10.57±0.52c | 13.54±0.54b | 19.75±0.94a | 15.60±1.06b |
| Total nitrogen (g·kg⁻¹) | 0.70±0.04c | 1.02±0.09b | 1.29±0.02a | 1.06±0.03b |
| Total phosphorus (g·kg⁻¹) | 0.79±0.04a | 0.79±0.01a | 0.79±0.03a | 0.80±0.05a |
| Total potassium (g·kg⁻¹) | 22.78±0.14a | 22.55±0.38a | 23.28±0.37a | 22.96±0.17a |
| Alkaline hydrolyzable nitrogen (mg·kg⁻¹) | 37.15±3.37c | 38.91±1.17bc | 50.37±2.14a | 45.97±2.11ab |
| Available phosphorus (mg·kg⁻¹) | 9.49±0.68b | 14.91±1.10a | 13.65±1.35a | 15.96±0.61a |
| Available potassium (mg·kg⁻¹) | 227.09±3.82c | 277.43±12.55a | 243.32±6.58bc | 265.76±7.97ab |
| Yield（t·hm^-2^） | 46.05±0.55b | 60.05±1.25a | 62.55±1.08a | 46.35±0.51b |

Note:The significance of differences was determined by the letter marking method (p < 0.05). Different lowercase letters in the same column indicate statistically significant differences among treatments. CF, chemical fertilizer alone; T1, sheep manure + chemical fertilizer; T2, biochar + chemical fertilizer; T3, corn stover + chemical fertilizer.

## 1.2 Supplementary Figures

**Supplementary Figure 1.** Coverage curves of fungal OTUs in potato rhizosphere soil under different fertilization treatments. CF, chemical fertilizer alone; T1, sheep manure + chemical fertilizer; T2, biochar + chemical fertilizer; T3, corn stover + chemical fertilizer.

|   a |   b |
| --- | --- |

**Supplementary Figure 2.** Chao index (a) and Shannon index (b) of fungal communities in potato rhizosphere soil under different fertilization treatments. CF, chemical fertilizer alone; T1, sheep manure + chemical fertilizer; T2, biochar + chemical fertilizer; T3, corn stover + chemical fertilizer. (The significant levels are indicated by asterisk:* p < 0.05; ** p < 0.01;*** p < 0.001)

**Supplementary Figure 3.** Relative abundance of fungal phyla in potato rhizosphere soil under different fertilization treatments. CF, chemical fertilizer alone; T1, sheep manure + chemical fertilizer; T2, biochar + chemical fertilizer; T3, corn stover + chemical fertilizer.

**Supplementary Figure 4.** Relative abundance of fungal genera in potato rhizosphere soil under different fertilization treatments. CF, chemical fertilizer alone; T1, sheep manure + chemical fertilizer; T2, biochar + chemical fertilizer; T3, corn stover + chemical fertilizer.

**Supplementary Figure 5.** PCoA analysis of fungal community structure in potato rhizosphere soil under different treatments based on Bray-Curtis distance at the OTU level. CF, chemical fertilizer alone; T1, sheep manure + chemical fertilizer; T2, biochar + chemical fertilizer; T3, corn stover + chemical fertilizer.

**Supplementary Figure 6.** LEfSe analysis of fungal communities in potato rhizosphere soil under different fertilization treatments (LDA > 3.8, p < 0.05). CF, chemical fertilizer alone; T1, sheep manure + chemical fertilizer; T2, biochar + chemical fertilizer; T3, corn stover + chemical fertilizer.

**Supplementary Figure 7.** Spearman correlation heatmap between fungal genera and soil physicochemical properties, as well as crop yield, in potato rhizosphere soil under different fertilization treatments. SOM, Soil organic matter; TN, total nitrogen; TP, total phosphorus; TK, total potassium; AN, alkaline hydrolyzable nitrogen; AP, available phosphorus; AK, available potassium. CF, chemical fertilizer alone; T1, sheep manure + chemical fertilizer; T2, biochar + chemical fertilizer; T3, corn stover + chemical fertilizer. (The significant levels are indicated by asterisk:* *p* < 0.05; ** *p* < 0.01;*** *p* < 0.001)

**Supplementary Figure 8.** Redundancy analysis (RDA) of fungal community at genus level and soil environmental factors in potato rhizosphere soil. SOM, Soil organic matter; TN, total nitrogen; TP, total phosphorus; TK, total potassium; AN, alkaline hydrolyzable nitrogen; AP, available phosphorus; AK, available potassium. CF, chemical fertilizer alone; T1, sheep manure + chemical fertilizer; T2, biochar + chemical fertilizer; T3, corn stover + chemical fertilizer.
